# Zika Virus Induces an Atypical Tripartite Unfolded Protein Response with Sustained Sensor and Transient Effector Activation and a Blunted BiP Response

**DOI:** 10.1128/mSphere.00361-21

**Published:** 2021-06-09

**Authors:** Mohammed Mufrrih, Biyao Chen, Shiu-Wan Chan

**Affiliations:** a Faculty of Biology, Medicine and Health, School of Biological Sciences, The University of Manchester, Manchester, United Kingdom; University of Michigan—Ann Arbor

**Keywords:** Zika virus, unfolded protein response, endoplasmic reticulum stress, virus-host interaction, host-pathogen interaction, integrated stress response, BiP, RNA virus, flavivirus

## Abstract

To study how the Zika virus (ZIKV) interacts with the host unfolded protein response (UPR), we undertook a kinetics study. We show that ZIKV infection triggers an atypical tripartite UPR in A549 cells involving transient activation of the effectors X-box-binding protein 1, activating transcription factor 4 (ATF4), CCAAT enhancer-binding protein-homologous protein, and growth arrest and DNA damage-inducible protein 34 during early infection and sustained activation of all three UPR sensors: RNA-activated protein kinase-like endoplasmic reticulum-resident kinase (PERK), inositol-requiring kinase-1α (IRE1α), and ATF6. Sustained phosphorylation of the eukaryotic translation initiation factor 2α and rRNA degradation coincide with host translational shutoff, cell lysis, and virus release during late infection. We show a blunted response of the master negative regulator, the immunoglobulin heavy-chain-binding protein (BiP), by chemical UPR inducers, and we show that ZIKV suppresses BiP transcription and translation, suggesting that it may be necessary to blunt the BiP response to sustain UPR sensor activation. The PERK inhibitor GSK2606414 alone has no effects but synergizes with the ATF6 inhibitor Ceapin-A7 to inhibit early and late infection, whereas Ceapin-A7 alone inhibits late infection. Likewise, 4-phenylbutyric acid inhibits ZIKV replication by attenuating the PERK and ATF6 pathways and potentiating the IRE1α pathway, suggesting that ZIKV infection is differentially and temporally regulated by different UPR arms. ZIKV infection is inhibited by pretreatment of chemical UPR inducers but is refractory to the inhibitory activity of chemical inducers once infection has been established, suggesting that ZIKV has anti-UPR mechanisms that may be able to modulate and co-opt the UPR in its life cycle.

**IMPORTANCE** The Zika virus originates from Africa and Asia but is emerging in other parts of the world. It usually causes an asymptomatic or mild, acute infection but can cause serious neurological complications, such as microcephaly and Guillain-Barré syndromes. Therefore, there is a pressing need for an antiviral. Viruses are obligative parasites and are dependent on the hosts for their propagation. As a result, we can target viruses by targeting host dependency. The host unfolded protein response is a cellular homeostatic response to stresses but can also be triggered by virus infections. We show here that Zika virus infection can cause stress and trigger the unfolded protein response. The Zika virus is able to manipulate, subvert, and co-opt the host unfolded protein response to aid its own replication. Understanding host dependency is important in the quest of a new class of antivirals called host-targeting agents.

## INTRODUCTION

Zika virus (ZIKV) is a vector-borne flavivirus transmitted by *Aedes* mosquito species (1). Its single-stranded, positive-sense RNA genome is translated into a single polyprotein, which is then cleaved by host and viral proteases into 10 proteins: capsid, premembrane, envelope (E), nonstructural 1 (NS1), NS2A, NS2B, NS3, NS4A, NS4B, and NS5 (2). ZIKV infection is mostly asymptomatic or mild but can result in neurological complications such as microcephaly and Guillain-Barré syndrome (3).

The unfolded protein response (UPR) is a tripartite, cellular adaptive response for restoring endoplasmic reticulum (ER) homeostasis in response to the presence of misfolded or unfolded proteins in the ER (4). The UPR is triggered by distraction of the master negative regulator, immunoglobulin heavy-chain-binding protein (BiP), from the three proximal UPR sensors: RNA-activated protein kinase (PKR)-like ER-resident kinase (PERK), inositol-requiring kinase 1α (IRE1α), and activating transcription factor 6 (ATF6), culminating in transcriptional and translational events to promote protein folding, global protein synthesis inhibition, and potentiation of ER-associated degradation (ERAD) to restore ER homeostasis (5, 6). IRE1α and PERK are activated by oligomerization and phosphorylation (7, 8). Phosphorylated IRE1α exhibits endonuclease activity to cleave the X-box-binding protein 1 (XBP1) by unconventional splicing into a transcription factor to transactivate genes involved in the UPR, ERAD and lipid biosynthesis (9). PERK is one of four mammalian eukaryotic initiation factor 2α (eIF2α) kinases that are activated by different stimuli but converge onto the phosphorylation of eIF2α at serine 51 to trigger the integrated stress response (ISR) (10). Phosphorylation of eIF2α causes global protein synthesis inhibition but upregulates translation of ATF4 which transactivates the CCAAT-enhancer-binding protein homologous protein (CHOP) and the growth arrest and DNA damage-inducible protein 34 (GADD34) (11). GADD34 negatively regulates the ISR by recruiting protein phosphatase 1 (PP1) to dephosphorylate eIF2α. Activated ATF6α migrates to the Golgi apparatus, where it is cleaved by site 1 protease and site 2 protease into an N-terminal 50-kDa fragment (p50nATF6), which then migrates to the nucleus to transactivate a subset of UPR and ERAD genes (12, 13).

Increasing evidence suggests a role for the UPR in virus infection (14, 15). Integrative *in silico* transcriptome analysis, whole-genome microarrays, and transcriptomic analysis (RNA-seq) have predicted activation of UPR genes in ZIKV-infected neural and fetal astrocytic cells and hepatocytes (16–18). The PERK and IRE1α pathways have been implicated in pathogenesis in mouse models of ZIKV infection, but results regarding the exact UPR signaling are contradictory (19–23). Because mice are refractory to ZIKV infection, infection of mice relies on the use of unnatural routes of intracranial and intraplacental injection or the use of immunocompromised knockout mice. It is, therefore, important to study ZIKV-induced UPR in immunocompetent human cells. The use of cultured human cells will also allow us to obtain a dynamic picture of virus-host interactions, which is difficult to achieve using mouse models. Thus far, attempts to demonstrate the UPR at the cellular level using human lung epithelial and neuronal cell lines, hepatocytes, neuronal stem cells and progenitor cells, primary human astrocytes, and human brain tissues and placental trophoblasts mainly focused on single-time-point detection of UPR effectors and selected UPR molecules, with disparate results (19–21, 24–29). An ATF6 activator, molecule 147, inhibited Dengue virus and ZIKV replication, but its inhibitory activity is independent of ATF6 activation in Dengue virus (30). Another inhibitor, CP26, which activates the UPR by inhibiting ERAD, inhibited Dengue virus and ZIKV infection, whereas another study demonstrated that ZIKV was not sensitive to ERAD gene knockout (31, 32). Hence, the exact nature of UPR signaling and its dynamic interaction with ZIKV is still far from clear. We, therefore, sought to study the kinetics of the UPR during an acute ZIKV infection and how ZIKV intertwines with the host UPR to facilitate virus replication. ZIKV infects a wide range of cell types, including human lung epithelial cells and fibroblasts and primary tree shrew lung cells (33–36). Increasing evidence suggests lung tropism of ZIKV. ZIKV has been detected in lung tissues of neonates and experimentally inoculated tree shrews and bats and was associated with lung pathologies (37–39). ZIKV replicates successfully in human lung implants in mice (40). Therefore, we expanded UPR study in a lung epithelial cell line, A549, because it supports robust and cytopathic ZIKV replication and is a common model in flavivirus studies.

## RESULTS

### ZIKV triggers a tripartite UPR in the early phase of infection.

**FIG 1:**
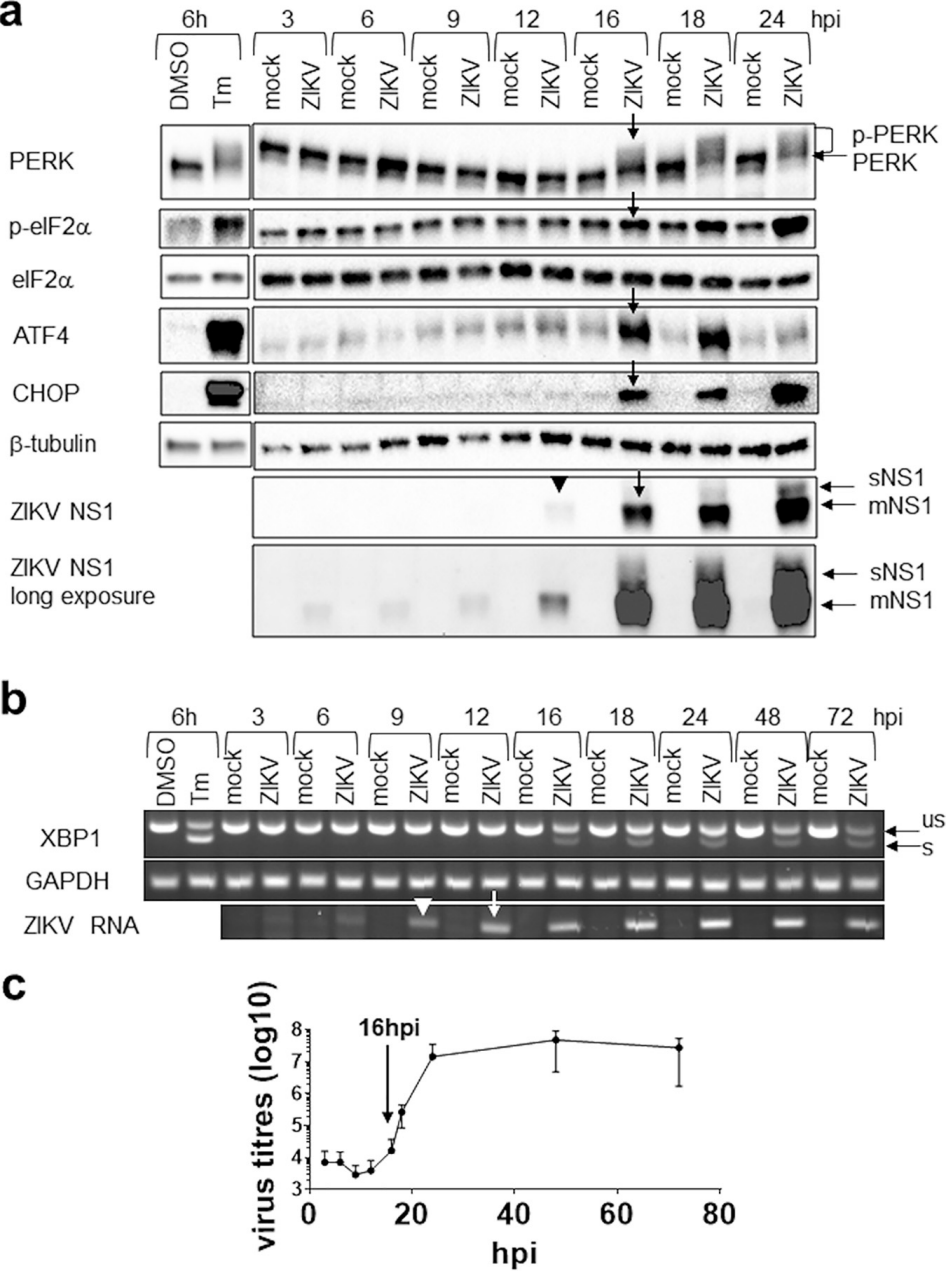
Zika virus replication triggers the unfolded protein response. A549 cells were mock infected or infected with the Zika virus (ZIKV) at an MOI of 1 for the indicated time or treated with 2 μg/ml tunicamycin (Tm) or DMSO solvent control for 6 h. (a) Western blots of the unfolded protein response (UPR) molecules. Results presented here are representative of two or three independent repeats. p-PERK is represented by slower-migrating bands. A long-exposure blot of the NS1 is also presented to reveal the weak signals at early time points. sNS1, soluble NS1; mNS1, membrane NS1. (b) Agarose gel electrophoresis of RT-PCR fragments showing unspliced (us) and spliced (s) XBP1 and ZIKV RNA. Presented is a representative blot from the same infection of three independent repeats. (c) Virus titers. Data are means ± standard deviations (SD) from three independent repeats. Arrowhead indicates the time point at which virus translation and replication started, and arrow indicates the time point at which virus translation, replication, and production surged and the UPR was induced. hpi, hours postinfection.

To understand the relationship of the UPR to the life cycle of ZIKV, we sought to study the kinetics of the UPR during early phase of ZIKV infection (3 to 24 h postinfection [hpi]) using a multiplicity of infection (MOI) of 1. Replication and a translation surge at 12 to 16 hpi preceded or coincided with UPR induction, indicating that the UPR is triggered by virus translation and replication (Fig. 1). PERK phosphorylation, commonly represented by slower-migrating bands, was detected from 16 hpi onward together with increased levels of phospho-eIF2α, ATF4, and CHOP, indicating induction of the ISR. XBP1 splicing was detected at 16 hpi (Fig. 1b). Together, these results suggest that early ZIKV infection triggers a UPR.

### Sustained UPR sensor activation and transient effector responses in the late phase of infection.

**FIG 2:**
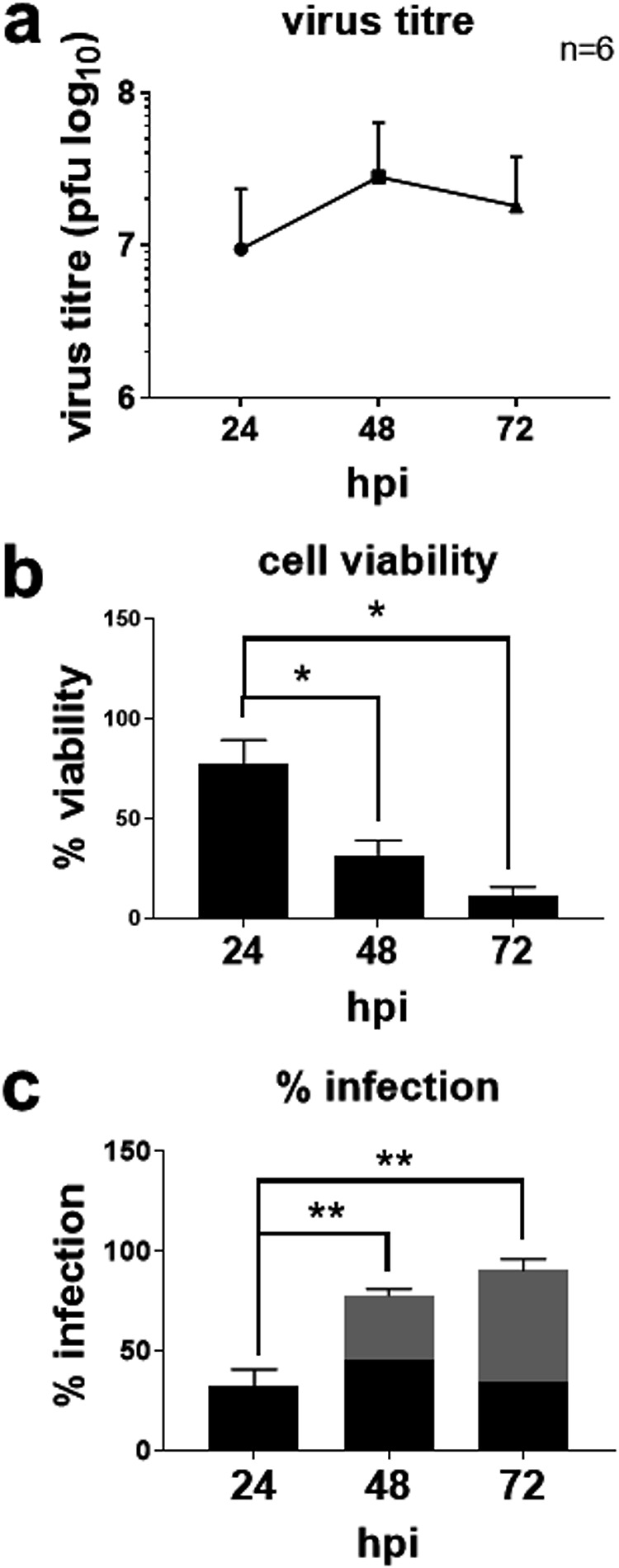
Time course of Zika virus infection. A549 cells were infected with Zika virus at an MOI of 1 for 24, 48, and 72 h postinfection (hpi). (a) Plaque assay data were analyzed by one-way ANOVA, and virus titers are presented as means and SD for six independent repeats. (b) Cell viability data were analyzed by one-way ANOVA and expressed as a percentage of the mock-infected control at the same time point, which is set as 100%. Data are means and SD for three independent repeats. (c) Percent infection, presented as means and SD for three independent repeats. Black bars represent percent infection in live cells. Gray bars represent the total percent infection in live and dead cells. Significance of the difference is represented by * (*P* < 0.05) and ** (*P* < 0.01).

Following a sharp rise from 16 hpi, the virus titer started to plateau from 24 hpi, peaked at 48 hpi, and slightly decreased at 72 hpi (Fig. 1c and 2a). The virus titer at 24 hpi was associated with 78% cell viability without visible cell death and 30% infected cells (Fig. 2b and c). High virus titers at 48 hpi and 72 hpi were associated with 68% and 89% cell death and 78% and 90% infection, suggesting that the majority of the viruses is released after 24 hpi up to 72 hpi. Note that about half of the live cells remained uninfected at all three time points, presumably due to establishment of an antiviral state by a paracrine interferon (IFN) effect (41).

**FIG 3:**
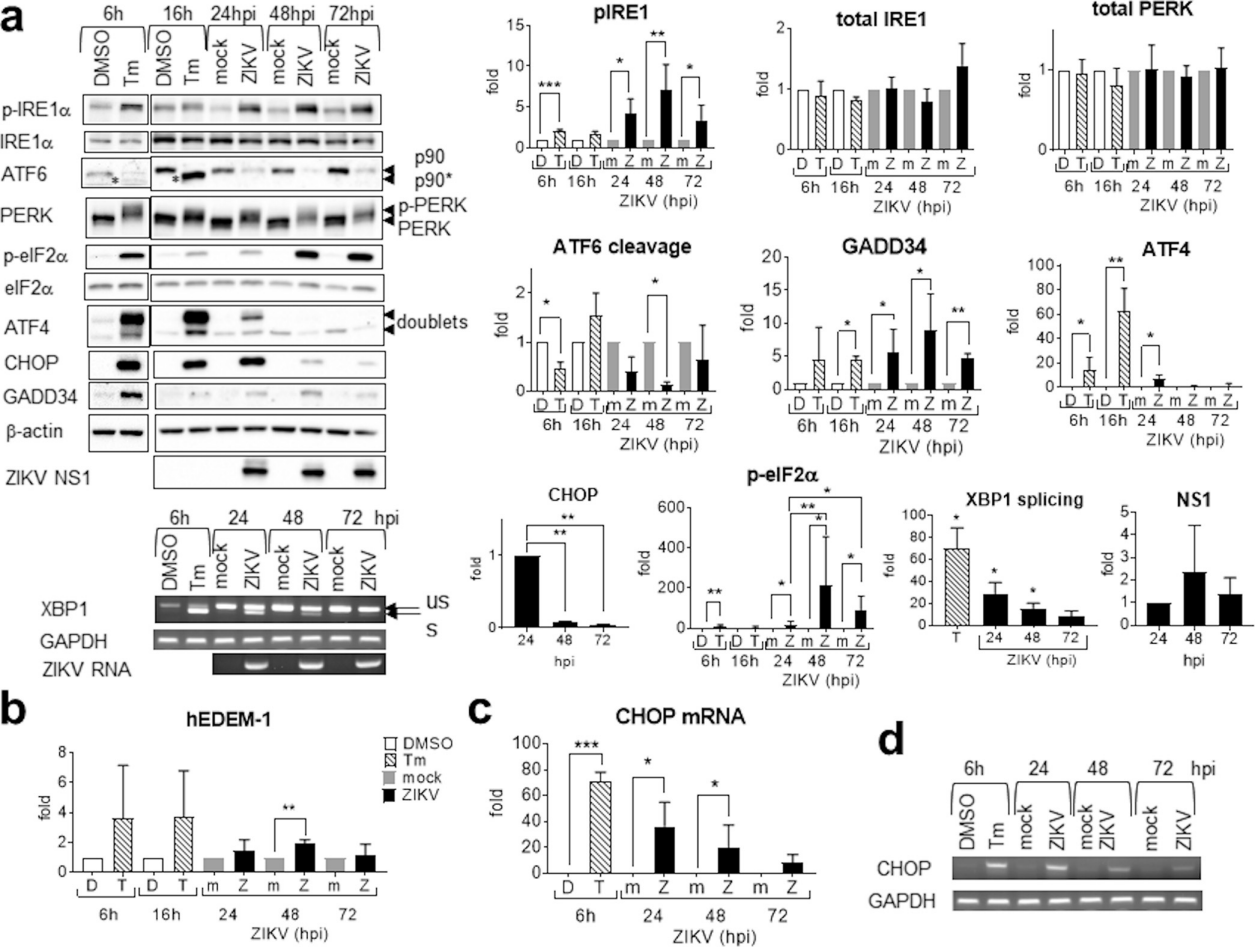
Zika virus triggers sustained activation of the unfolded protein response sensors. A549 cells were mock infected or infected with UV-inactivated ZIKV or live ZIKV at an MOI of 1 for 24, 48, and 72 h or treated with 2 μg/ml tunicamycin (Tm) or DMSO control for 6 and 16 h. (a) Western blots (top) and agarose gels (bottom) of the UPR molecules. On the left is a representative blot from the same infection of three independent repeats. Protein bands were quantified, normalized against either the internal control β-actin (for PERK, ATF6, ATF4, CHOP, GADD34, and NS1), total IRE1α (for p-IRE1α), or total eIF2α (for p-eIF2α), and expressed as a ratio of the mock-infected or solvent controls at the same time point, which were set as 1. p90* indicates unglycosylated ATF6. Because Tm inhibits glycosylation, newly synthesized p90ATF6 is unglycosylated and appears as a faster-migrating p90* band (12). p-PERK is represented by slower-migrating bands. ATF4 migrates as doublets. Agarose gel electrophoresis of RT-PCR fragments showing unspliced (us) and spliced (s) XBP1. Percent splicing is expressed as percent of sXBP1 relative to total XBP1 (sXBP1+usXBP1), and statistical analysis of XBP1 splicing included data from Fig. 1b. Data on the right are means and SD for two to four independent repeats. (b) Real-time RT-qPCR of hEDEM1. (c and d) Real-time RT-qPCR (c) and agarose gel electrophoresis (d) of CHOP mRNAs. Data were normalized against an endogenous control GAPDH mRNA and are expressed as a ratio of either the mock-infected control at the same time point or solvent control, which was set as 1. Data are means and SD for three independent infections. *, *P* < 0.05; **, *P* < 0.01; ***, *P* < 0.001. hpi, hours postinfection.

We then sought to study the kinetics of the UPR during the late phase of infection (24 to 72 hpi) using an MOI of 1. The level of phospho-IRE1α increased significantly at 24 hpi, peaked at 48 hpi, and remained high at 72 hpi, whereas the level of total IRE1α remained unchanged (Fig. 3a). In contrast, XBP1 splicing was transient, with maximal splicing occurring at 24 hpi followed by a modest but significant upregulation of its effector, the human ERAD-enhancing α-mannosidase-like protein 1 (hEDEM1), at 48 hpi, indicating ERAD induction (Fig. 3a and b). The cleaved product of ATF6, p50nATF6, is very unstable and difficult to detect; therefore, ATF6 cleavage is commonly represented by the disappearance of the full-length p90ATF6 (12). p90ATF6 disappearance was detected at 24 hpi, peaked at 48 hpi, and sustained until 72 hpi (Fig. 3a). The level of phospho-PERK increased from 16 to 24 hpi, peaked at 48 hpi, and remained high at 72 hpi, whereas the level of total PERK remained unchanged (Fig. 1a and 3a). Similarly, the level of phospho-eIF2α increased from 16 hpi to 20-fold at 24 hpi and then dramatically to 215- and 93-fold at 48 and 72 hpi. In contrast, downstream ISR was transient and blunted in the face of sustained phosphorylation of eIF2α. ATF4 and CHOP expression peaked at 16 and 24 hpi, respectively, and then sharply declined from 24 hpi to undetectable and very low levels at 48 hpi. The CHOP mRNA level, however, was significantly increased at 24 and 48 hpi, suggesting that the sharp decline in CHOP expression at 48 hpi is due to a translational block (Fig. 3c and d). GADD34 expression was modestly upregulated at 24, 48, and 72 hpi with peak expression at 48 hpi (Fig. 3a). Together, our results showed that transient UPR effectors are associated with the early phase of infection and blunted in the face of sustained UPR sensor activation.

**FIG 4:**
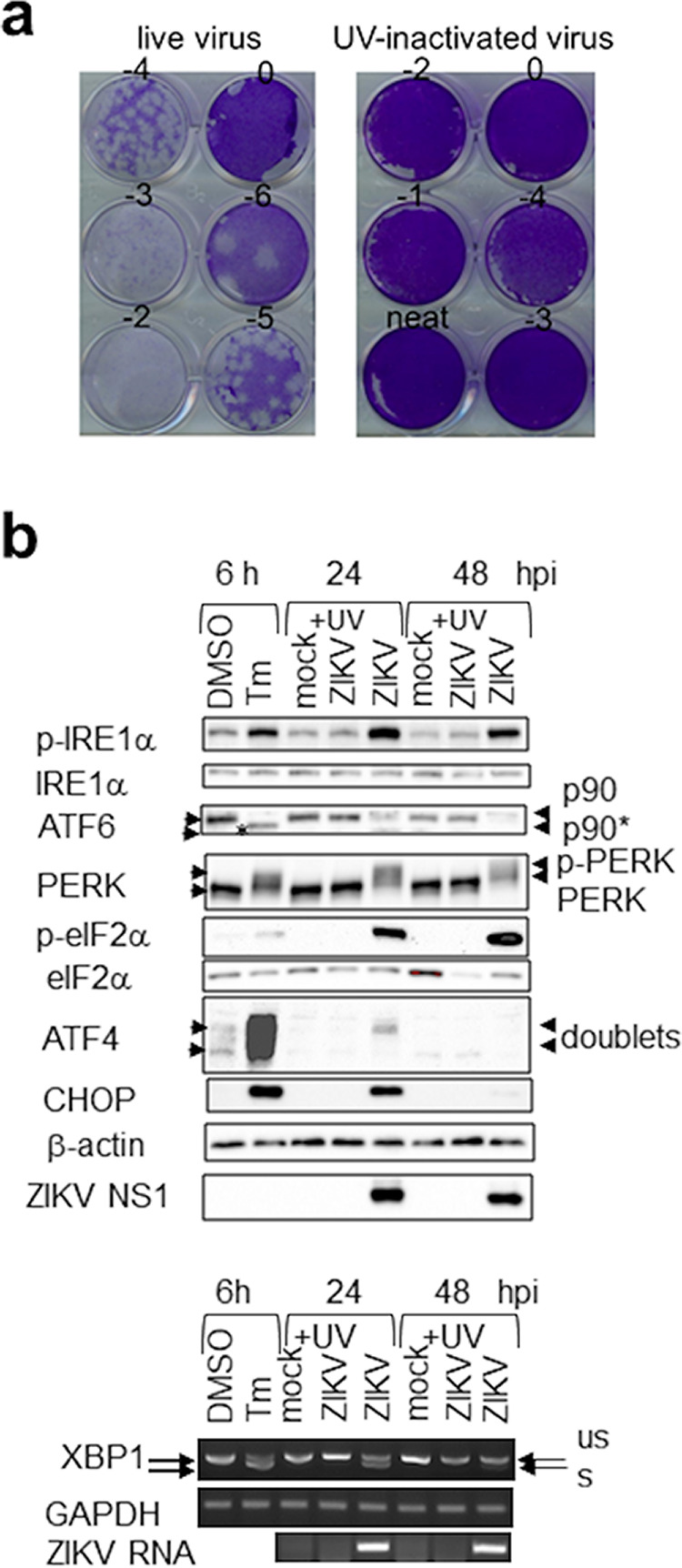
UV-inactivated virus does not induce the unfolded protein response. A549 cells were mock infected or infected with UV-inactivated or live ZIKV at an MOI of 1 for 24 and 48 h or treated with 2 μg/ml tunicamycin (Tm) or DMSO control for 6 h. (a) Plaque assay showing the absence of infectious virus in UV-inactivated virus samples compared to live virus samples. (b) Western blots (top) and agarose gels (bottom) of the UPR molecules. Presented is a representative of UV-inactivated samples from the same infection of two independent repeats. p90* indicates unglycosylated ATF6. p-PERK is represented by slower-migrating bands. ATF4 migrates as doublets. us, unspliced XBP1; s, spliced XBP1.

To exclude the possibility that it was the supernatant or virus attachment rather than virus infection that triggered the UPR, we used UV-inactivated ZIKV. Virus inactivation was confirmed using plaque assay and the absence of viral protein expression or replication (Fig. 4). In contrast to the tunicamycin (Tm) control and live ZIKV infection and similar to the mock-infected control, UV-inactivated ZIKV did not induce phosphorylation of PERK, eIF2α, and IRE1α, ATF6 cleavage, XBP1 splicing, or ATF4 and CHOP expression at 24 and 48 hpi, confirming that UPR activation requires active ZIKV infection and replication (Fig. 4b).

### ATF6 plays a role in the late phase of infection.

**FIG 5:**
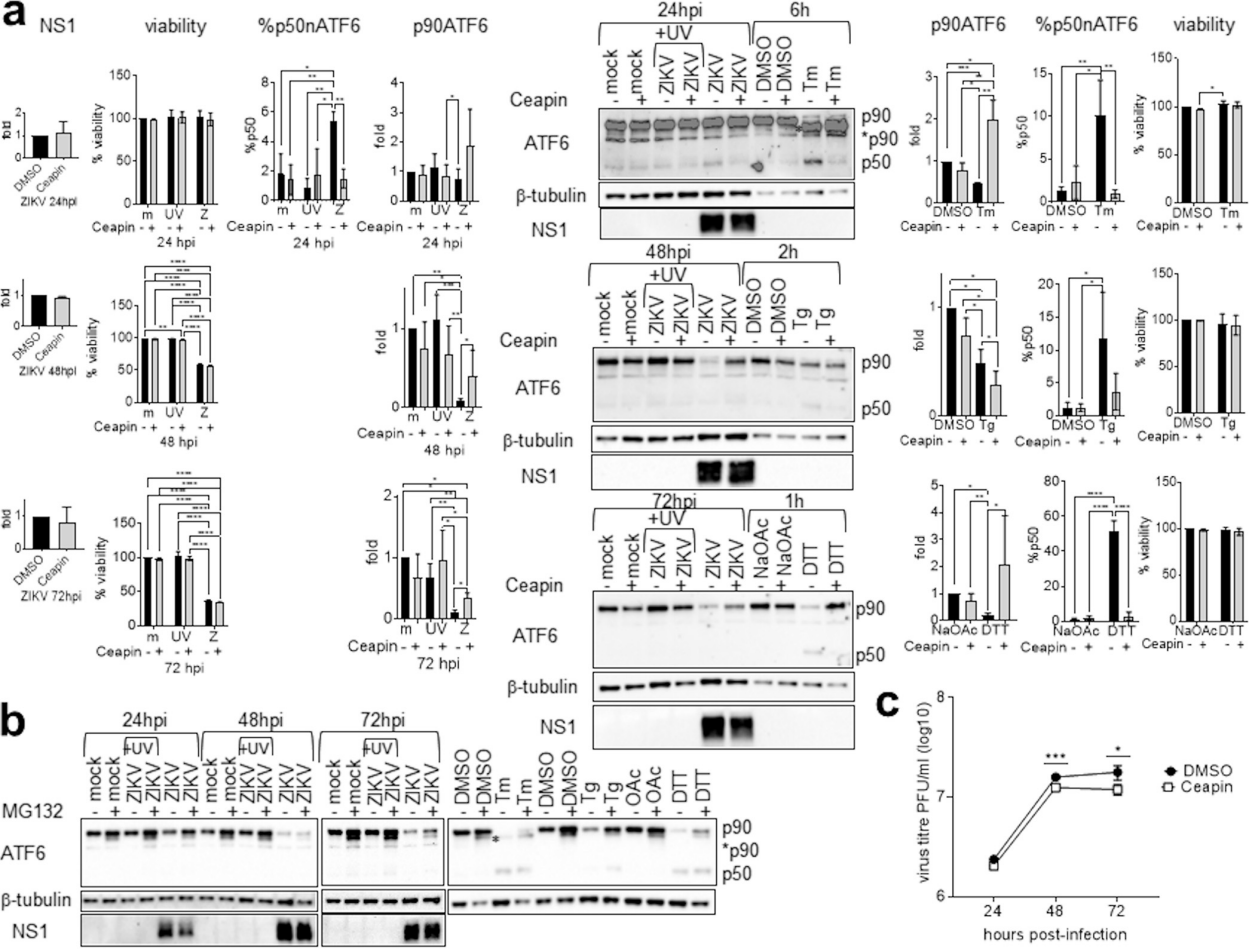
Ceapin-A7 inhibits ATF6 cleavage at the late phase of infection. A549 cells were mock infected or infected with UV-inactivated ZIKV or live ZIKV at an MOI of 1 for 24, 48, and 72 h in the presence of chemical inhibitor or DMSO solvent control. UPR inducer controls include A549 cells treated with 2 μg/ml tunicamycin (Tm) or DMSO solvent control for 6 h, 400 nM thapsigargin (Tg) or DMSO solvent control for 2 h, and 1 mM DTT or sodium acetate (NaOAc) solvent control for 1 h. (a) Western blots of ATF6 in the presence or absence of 6 μM Ceapin-A7. Ceapin-A7 was added 2 hpi and 1 h before addition of UPR inducer controls. Presented is a representative from the same infection of three independent repeats. The blot was overexposed to reveal the p50nATF6. Protein bands were quantified using low-exposure bands, normalized against an internal control β-tubulin, and expressed as a ratio to either mock-infected or solvent controls without Ceapin-A7 at the same time point, which were set as 1. Percentage of cleaved p50nATF6 is expressed relative to the total p90ATF6 (p90)+p50nATF6 (p50) bands. Data are means and SD for three independent repeats. p90* indicates unglycosylated ATF6. m, mock; UV, UV-inactivated ZIKV; Z, ZIKV. Cell viability data were analyzed by two-way ANOVA and are presented as means and SD for three independent repeats, expressed as a percentage of mock-infected or solvent controls without Ceapin-A7 at the same time point, which were set as 100%. (b) Western blots of ATF6 in the presence or absence of 10 μM MG132. MG132 was added 4 h before harvest from infected cells and 4 h before addition of UPR inducer controls. Presented is a representative from the same infection of two independent repeats. Cell viability data are not shown. (c) Plaque assay data were analyzed by paired *t* test, and virus titers are presented as means and SD for three independent repeats. *, *P* < 0.05; **, *P* < 0.01; ***, *P* < 0.001; ****, *P* < 0.0001. hpi, hours postinfection.

It is still inconclusive whether ZIKV infection activates ATF6. We therefore sought to study the role of ATF6 in ZIKV infection. p50nATF6 is very unstable and difficult to detect, as exemplified by the detection of only 10% p50nATF6 in Tm- and thapsigargin (Tg)-treated samples, although the use of a potent ATF6 inducer, dithiothreitol (DTT), increased p50nATF6 to 50% (Fig. 5a). Nevertheless, we detected a significant level of p50nATF6, 5%, at 24 hpi, which was completely inhibited by a specific ATF6 cleavage inhibitor, Ceapin-A7, at a noncytotoxic level, confirming ATF6 activation in ZIKV infection (42). However, no p50nATF6 was detected at 48 and 72 hpi despite significant p90ATF6 disappearance. It was possible that we could not detect p50nATF6 due to accelerated degradation at late phase of infection. On the other hand, p90ATF6 disappearance could be due to ERAD degradation, caspase activity, or translational shutoff. The absence of any newly cleaved bands showed that p90ATF6 disappearance was unlikely a result of caspase cleavage. A noncytotoxic level of the proteasome inhibitor MG132 was able to restore a basal level of p90ATF6 but was unable to inhibit p90ATF6 disappearance and cleavage in cells infected with ZIKV or treated with chemical UPR inducers, indicating that ERAD degradation is not responsible for p90ATF6 disappearance (Fig. 5b). In contrast, a noncytotoxic level of Ceapin-A7 significantly inhibited p90ATF6 disappearance at 48 and 72 hpi and also in Tm and DTT controls, confirming that p90ATF6 disappearance at 48 and 72 hpi is a result of ATF6 cleavage (Fig. 5a). Because inhibition of p90ATF6 disappearance was partial at 48 and 72 hpi in contrast to complete inhibition in Tm and DTT control samples, it remained possible that host translational shutoff accounted for some of the p90ATF6 disappearance, given that p90ATF6 is unstable, with a short half-life of 2 h (12). Ceapin-A7 treatment resulted in a low-level reduction in virus titer at 48 and 72 hpi, which may be due to incomplete inhibition of ATF6 activation and/or additional dependence of ZIKV replication on the PERK and IRE1α pathways (Fig. 5c). Nevertheless, the significant reduction in virus titers indicates a role of the ATF6 pathway in late phase of infection.

### PERK is not responsible for early eIF2α phosphorylation but could be the major kinase during late phase of infection.

**FIG 6:**
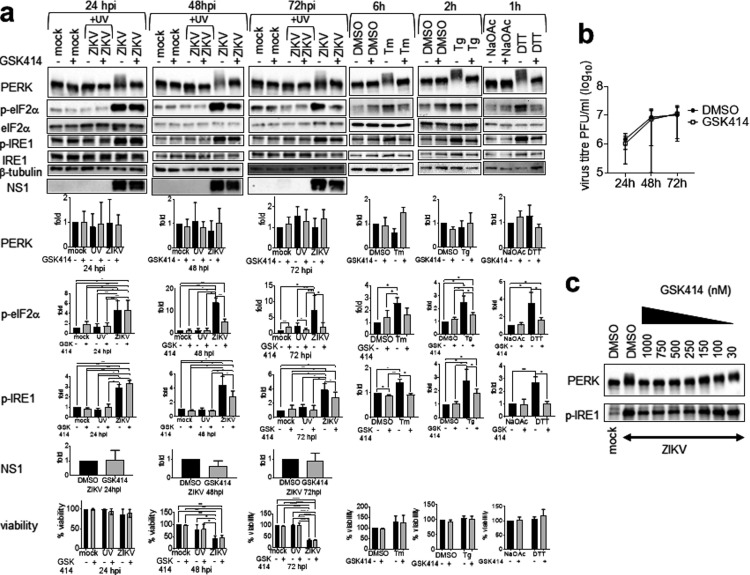
GSK2606414 inhibits PERK phosphorylation but not ZIKV yield. A549 cells were mock infected or infected with UV-inactivated ZIKV or live ZIKV at an MOI of 1 for 24, 48, and 72h in the presence of GSK2606414 (GSK414) or DMSO solvent control. UPR inducer controls include A549 cells treated with 2 μg/ml tunicamycin (Tm) or DMSO solvent control for 6 h, 400 nM thapsigargin (Tg) or DMSO solvent control for 2 h, and 1 mM DTT or sodium acetate (NaOAc) solvent control for 1 h. (a) Western blots of the UPR molecules in the presence or absence of 1 μM GSK414. GSK414 was added 2 hpi and 1 h before addition of UPR inducer controls. Presented is a representative of three independent repeats. Protein bands were quantified, normalized against either the internal control β-tubulin (for PERK and NS1), total IRE1α (for p-IRE1α), or total eIF2α (for p-eIF2α), and expressed as a ratio to either mock-infected or solvent controls without GSK414 at the same time point, which were set as 1. Data are means and SD for three independent repeats. p-PERK is represented by slower-migrating bands. Cell viability data were analyzed by two-way ANOVA and are presented as means and SD for three independent repeats, expressed as a percentage of mock-infected or solvent controls without GSK414 at the same time point, which were set as 100%. (b) Plaque assay data were analyzed by paired *t* test, and virus titers are presented as means and SD for four independent repeats. (c) Western blots showing p-PERK and p-IRE1α in ZIKV-infected cells in the presence of serial doses of GSK414. *, *P* < 0.05; **, *P* < 0.01; ****, *P* < 0.0001. hpi, hours postinfection.

eIF2α can be phosphorylated by one of four ISR kinases (10). To investigate the role of PERK in eIF2α phosphorylation, we used a PERK phosphorylation inhibitor, GSK2606414 (43). GSK2606414, at a noncytotoxic concentration, completely inhibited PERK phosphorylation at 24 to 72 hpi and by the chemical inducers Tm, Tg, and DTT without affecting total PERK levels, confirming that band shift is caused by PERK phosphorylation (Fig. 6a). Complete inhibition of PERK phosphorylation at 24 hpi did not inhibit eIF2α phosphorylation, suggesting that PERK is not the major kinase of eIF2α phosphorylation at 24 hpi. Complete inhibition of PERK phosphorylation led to significant 61% and 72% decreases of phospho-eIF2α at 48 hpi and 72 hpi, suggesting that PERK could be the major but not the only eIF2α kinase at the late phase of infection. Complete inhibition of PERK did not affect virus titers at 24 to 72 hpi, suggesting that PERK phosphorylation alone has no effects on virus replication (Fig. 6b). Surprisingly, GSK2606414 also partially inhibited IRE1α phosphorylation at 48 and 72 hpi, following the same time course of eIF2α phosphorylation inhibition (Fig. 6a). Titration of GSK2606414 could not uncouple its inhibitory effects on PERK and IRE1α phosphorylation (Fig. 6c).

### PERK and ATF6 play a synergistic role in ZIKV infection.

**FIG 7:**
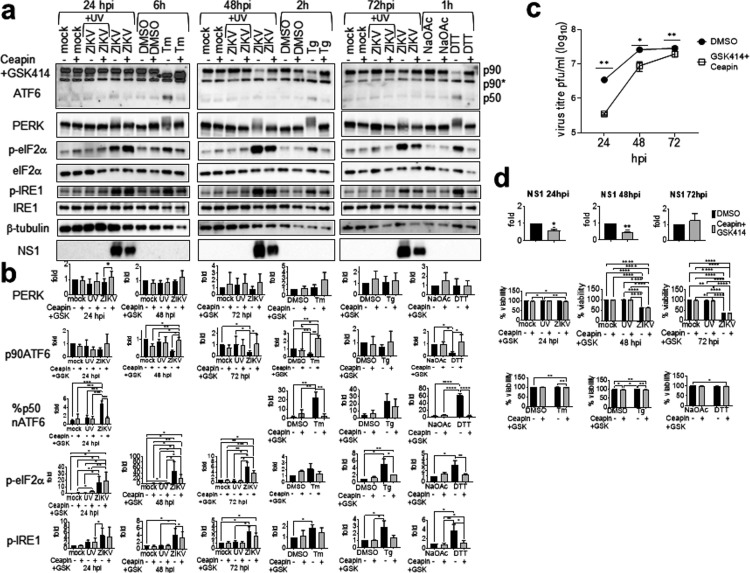
GSK2606414 and Ceapin-A7 synergistically inhibit ZIKV yields at early and late phases of infection. A549 cells were mock infected or infected with UV-inactivated ZIKV or live ZIKV at an MOI of 1 for 24, 48, and 72 h in the presence of 1 μM GSK2606414 (GSK414) and 6 μM Ceapin-A7 or DMSO solvent control. UPR inducer controls include A549 cells treated with 2 μg/ml tunicamycin (Tm) or DMSO solvent control for 6 h, 400 nM thapsigargin (Tg) or DMSO solvent control for 2 h, and 1 mM DTT or sodium acetate (NaOAc) solvent control for 1 h. (a) Western blots of the UPR molecules in the presence or absence of GSK414 and Ceapin-A7. GSK414 and Ceapin-A7 were added 2 hpi and 1 h before addition of UPR inducer controls. The blot was overexposed to reveal the p50nATF6. p90* indicates unglycosylated ATF6; p50, p50nATF6; p-PERK is represented by slower-migrating bands. (b) Protein bands were quantified using low-exposure bands, normalized against either internal control β-tubulin (for PERK and ATF6), total IRE1α (for p-IRE1α), or total eIF2α (for p-eIF2α), and expressed as a ratio to either mock-infected or solvent controls without GSK414 and Ceapin-A7 at the same time point, which were set as 1. Percent cleaved p50nATF6 (%p50ATF6) is expressed relative to the total p90ATF6 (p90)+p50nATF6 (p50) bands. Data are means and SD for three independent repeats. (c) Plaque assay data were analyzed by paired *t* test, and virus titers are presented as means and SD for three independent repeats. (d) NS1 bands from panel a, normalized against β-tubulin, were analyzed by one-sample *t* test and are presented as means and SD for three independent repeats. Cell viability data were analyzed by two-way ANOVA and are presented as means and SD for three independent repeats, expressed as a percentage of mock-infected or solvent controls without inhibitors at the same time point, which were set as 100%. *, *P* < 0.05; **, *P* < 0.01; ***, *P* < 0.001; ****, *P* < 0.0001. hpi, hours postinfection.

**FIG 8:**
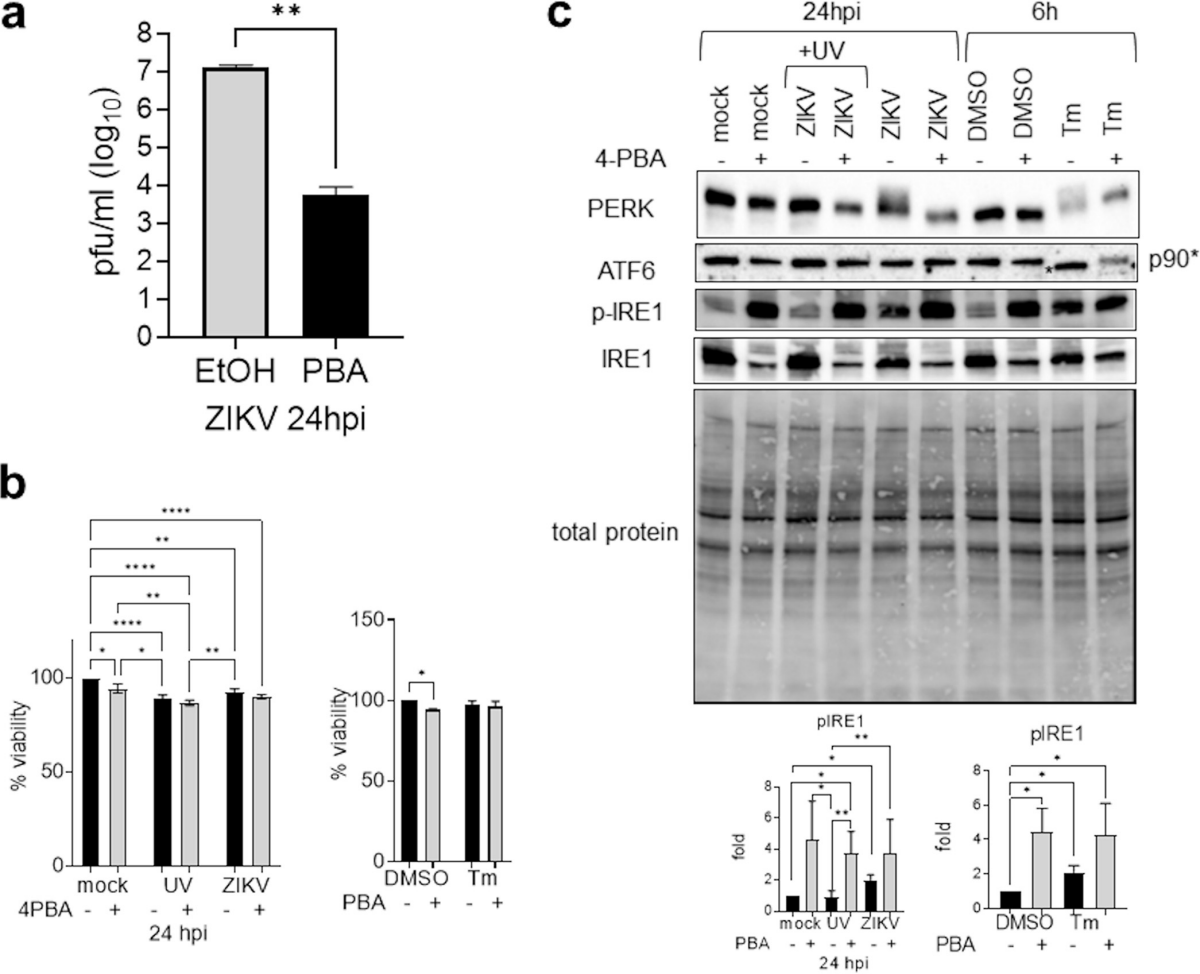
4-PBA inhibits Zika virus replication. A549 cells were mock infected or infected with UV-inactivated ZIKV or live ZIKV at an MOI of 1 for 24 h in the presence of 15 mM 4-phenylbutyric acid (4-PBA) or the solvent control ethanol (EtOH) or treated with the UPR inducer tunicamycin (Tm; 2 μg/ml) or DMSO solvent control for 6 h. 4-PBA was added 2 hpi and 1 h before addition of UPR inducer control. (a) Plaque assay data were analyzed by paired *t* test, and virus titers are presented as means and SD for three independent repeats. (b) Cell viability data were analyzed by two-way ANOVA and are presented as means and SD for three independent repeats, expressed as a percentage of mock-infected or solvent controls without inhibitors at the same time point, which were set as 100%. (c) Western blots of the UPR sensors in the presence or absence of 4-PBA. p90* indicates unglycosylated ATF6; p-PERK is represented by slower-migrating bands. Total protein was detected on TGX stain-free gels (Bio-Rad). p-IRE1α bands were normalized against total IRE1α and expressed as a ratio to either mock-infected or solvent controls without 4-PBA, which were set as 1. Data are means and SD for three independent repeats. *, *P* < 0.05; **, *P* < 0.01; ****, *P* < 0.0001. hpi, hours postinfection.

The early phase of ZIKV infection was not inhibited by GSK2606414 or Ceapin-A7. Ceapin-A7 inhibited a low-level but significant reduction of virus titer at the late phase of infection (Fig. 5c). Therefore, we sought to study cooperation of the two UPR arms in ZIKV infection. Combined use of GSK2606414 and Ceapin-A7 inhibited their targets similarly to use of each alone, and the combination was noncytotoxic (Fig. 7a, b, and d). GSK2606414 and Ceapin-A7 exhibited a synergistic inhibitory effect on ZIKV yields at 24 to 72 hpi, with maximal inhibition at 24 hpi (10-fold reduction in virus titer) (Fig. 7c). At 24 hpi, GSK2606414 caused complete inhibition of phospho-PERK but not phospho-eIF2α or phospho-IRE1α, showing that inhibition of virus titer at 24 hpi by GSK2606414 and Ceapin-A7 is due to synergistic effects of phospho-PERK and the ATF6 pathway (Fig. 6a and 7a). Inhibition of PERK phosphorylation and ATF6 cleavage by the ER stress inhibitor 4-phenylbutyrate (4-PBA) at a noncytotoxic level also resulted in a dramatic 3.74-log reduction of virus titer at 24 hpi (Fig. 8). Paradoxically, 4-PBA increased IRE1α phosphorylation concomitant with total IRE1α degradation, resulting in about a 2-fold increase in relative phospho-IRE1α level (Fig. 8c). Together, these results suggest that significant inhibition of ZIKV replication can be achieved by attenuating PERK and ATF6 activation and increased IRE1α phosphorylation at an early time of infection.

### eIF2α phosphorylation and rRNA degradation coincides with host translational shutoff, cell death, and virus release at a late phase of infection.

**FIG 9:**
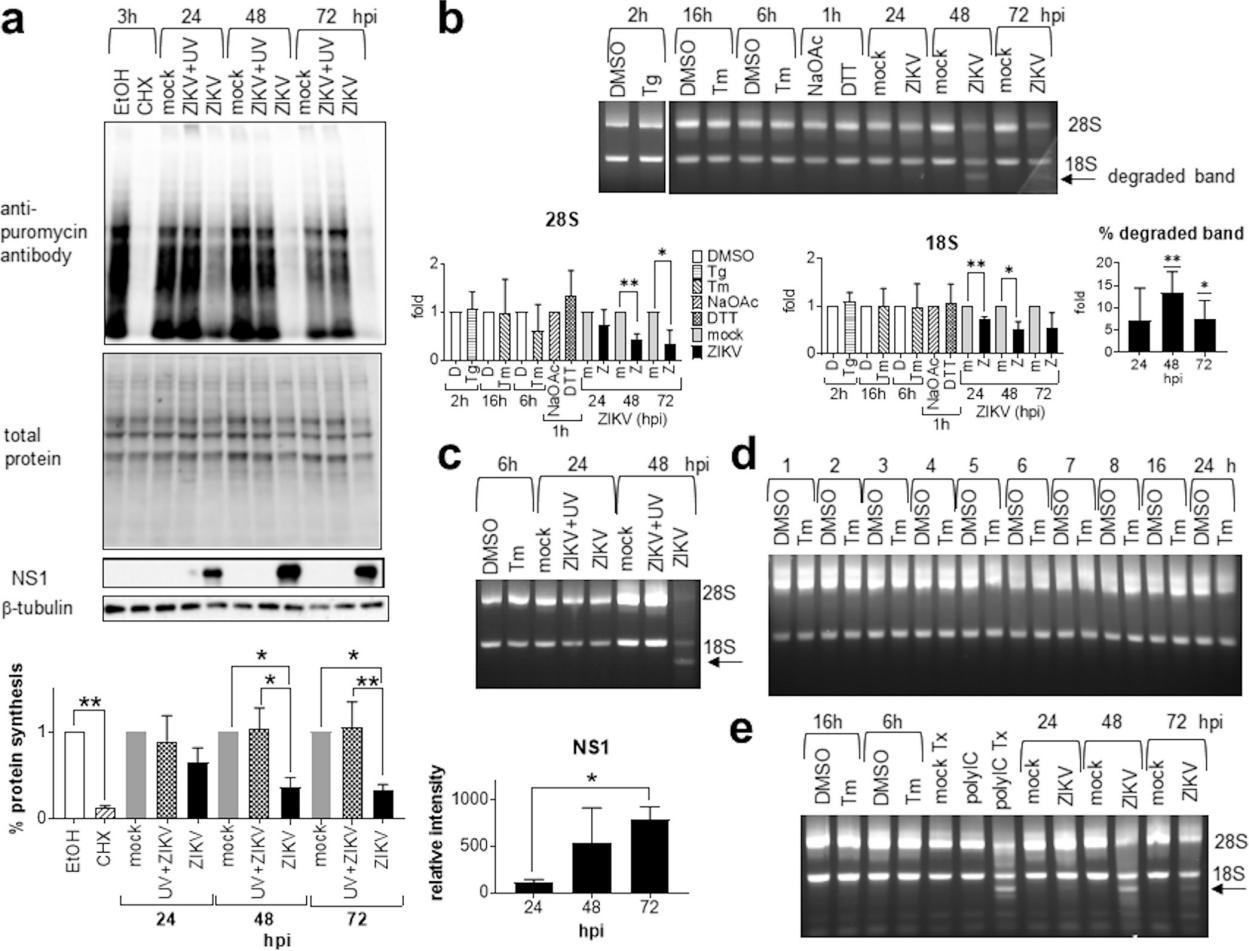
Zika virus causes host translational shutoff and rRNA degradation. (a) A549 cells were mock infected or infected with UV-inactivated ZIKV or live ZIKV at an MOI of 1 for 24, 48, and 72 h or treated with 2 μg/ml cycloheximide or EtOH solvent control for 3 h. Puromycin was added 1 h before harvesting for Western blotting. A Western blot of puromycin bands representing *de novo*-synthesized proteins is shown. Total protein was detected on TGX stain-free gels (Bio-Rad). Presented is a representative from the same infection of three independent repeats. Protein synthesis was measured by normalizing total puromycin bands against total proteins on the TGX gel loading control and expressed as a ratio to either mock-infected control at the same time point or solvent control, which were set as 1. Data are means and SD for three independent repeats. NS1 was quantified by normalizing the NS1 band on Western blots against total proteins on the TGX gel loading control, and the result was expressed as relative intensity. Data are means and SD for three independent repeats. (b to d) A549 cells were mock infected or infected with UV-inactivated ZIKV or live ZIKV at an MOI of 1 for 24, 48, and 72 h. UPR inducer controls include A549 cells treated with 2 μg/ml tunicamycin (Tm) or DMSO solvent control for 6 or 16 h, 400 nM thapsigargin (Tg) or DMSO solvent control for 2 h, and 1 mM DTT or sodium acetate (NaOAc) solvent control for 1 h. (b) Agarose gel electrophoresis of RNA showing the 28S and 18S rRNA bands and the degraded band (arrow). The 28S and 18S rRNAs were quantified and expressed as a ratio to either mock-infected control at the same time point or solvent control, which were set as 1. The degraded band was expressed as a ratio to total rRNA bands. Data are means and SD for three independent repeats. (c and d) Agarose gel electrophoresis of RNA showing the absence of 28S and 18S rRNA degradation in UV-inactivated ZIKV samples (c) and a Tm time course (d). (e) A549 cells were mock infected or infected with ZIKV at an MOI of 1 for 24, 48, and 72 h or treated with 2 μg/ml tunicamycin (Tm) or DMSO solvent control for 6 and 16 h, mock transfected (Tx), transfected with 2 μg poly(I·C) (polyIC Tx), or incubated with poly(I·C) without transfection (polyIC). Agarose gel electrophoresis of RNA showing the 28S and 18S rRNA bands and the degraded band (arrow). hpi, hours postinfection. *, *P* < 0.05; **, *P* < 0.01.

Phosphorylation of eIF2α inhibits global protein synthesis; therefore, we sought to study the effects of ZIKV infection on host translation (8). Compared with the protein synthesis inhibitor, cycloheximide, which inhibited 88% of protein synthesis, ZIKV but not UV-inactivated virus infection inhibited 34, 64, and 68% of protein synthesis at 24, 48, and 72 hpi, confirming that protein synthesis inhibition requires active virus replication (Fig. 9a).

Paradoxically, phosphorylation of eIF2α upregulates translation of ATF4 to launch the ISR in a negative feedback loop to restore ER homeostasis (10). However, ZIKV infection failed to induce ATF4 expression at 48 and 72 hpi despite dramatic increases in eIF2α phosphorylation (Fig. 3a). This led us to examine other possibilities of protein synthesis inhibition. ZIKV but not UV-inactivated virus infection induced a small degree of 28S and 18S rRNA degradation at 24 hpi, followed by dramatic degradation at 48 and 72 hpi with the appearance of a newly degraded band, which might explain inhibition of ATF4 translation at these time points even when eIF2α was phosphorylated (Fig. 9b and c). Degradation of both 28S and 18S rRNAs in ZIKV-infected cells argues against a regulated IRE1-dependent degradation (RIDD) mechanism, which involves only 28S rRNA degradation (44). The absence of RIDD was corroborated by the absence of rRNA degradation in a Tm time course and in Tg- and DTT-treated cells (Fig. 9b and d). In contrast, 28S and 18S rRNA degradation is a hallmark feature of the antiviral effector, RNase L (45). RNase L is activated by 2′,5′-oligoadenylate synthase, which is in turn activated by IFN. ZIKV-induced rRNA degradation shared similarity with the patterns generated by intracellular poly(I·C), a synthetic double-stranded-RNA (dsRNA) structural mimic commonly used to trigger the IFN response, suggesting that ZIKV infection may induce rRNA degradation via the IFN response (Fig. 9e) (46). Together these results suggest that eIF2α phosphorylation and rRNA degradation are associated with translational shutoff, which coincides with cell lysis, virus release, and spreading.

### A blunted BiP response in ZIKV infection.

**FIG 10:**
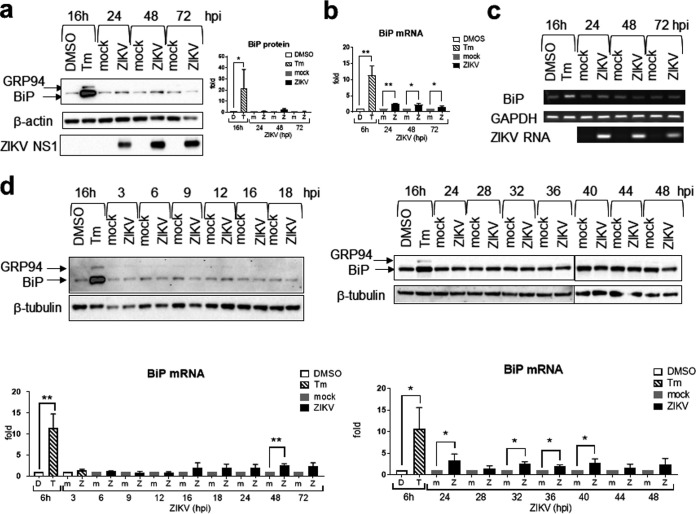
Zika virus triggers very modest BiP activation. A549 cells were mock infected or infected with ZIKV at an MOI of 1 for the indicated time or treated with 2 μg/ml tunicamycin (Tm) or DMSO solvent control for 16 h. (a) Western blots of BiP. Presented is a representative from the same infection of three independent repeats. The blot was overexposed to reveal the weak signals in infected samples. Protein bands from low exposure bands were quantified, normalized against a β-actin internal control, and expressed as a ratio to either a mock-infected control at the same time point or a solvent control, which were set as 1. Data on the right are means and SD for three independent repeats. (b) Real-time RT-qPCR of BiP mRNA. Data were normalized against an endogenous control GAPDH mRNA and are expressed as a ratio to either a mock-infected control at the same time point or a solvent control, which were set as 1. Data are means and SD for three independent infections. (c) Agarose gel electrophoresis showing RT-PCR fragments of BiP. Presented is a representative from the same infection of two independent repeats. (d) Western blots (top) and real-time RT-qPCR (bottom) of ZIKV infection at early time points and from 24 to 48 hpi. Western blots are representative of two or three independent repeats. Real-time RT-qPCR data were normalized against an endogenous control GAPDH mRNA and are expressed as a ratio to either a mock-infected control at the same time point or a solvent control, which were set as 1. Data are means and SD for three independent infections. *, *P* < 0.05; **, *P* < 0.01.

BiP is the master negative regulator of the UPR (6, 47). It is transcriptionally upregulated by the UPR effectors to restore ER homeostasis in a negative feedback loop. Sustained activation of the UPR sensors in ZIKV infection may imply a dysregulated BiP activity; therefore, we investigated BiP activation in ZIKV infection. Because BiP is under feedback regulation and single-time-point analysis would have missed transient activation, we ran multiple time courses. We failed to detect any significant increases of BiP expression at early (3 to 24 hpi) and late (24 to 72 hpi) phases of infection or any transient activation from 24 to 48 hpi (Fig. 10a and d). Because the anti-KDEL antibody also recognizes the glucose-regulated protein 94 (GRP94), another UPR effector, we could also detect a similar suppressive effect of ZIKV infection on GRP94. At the transcriptional level, significant but very modest 2.5-, 2.2-, and 1.6-fold increases of BiP mRNA were detected at 24, 48, and 72 hpi by real-time RT-qPCR but not by semiquantitative agarose gel RT-PCR (Fig. 10b and c). Similarly, using real-time RT-qPCR, very modest increases in BiP mRNA were detected during the early phase of infection (3 to 24 hpi) and between 24 and 48 hpi (Fig. 10d). Together, these results suggest a blunted BiP response during ZIKV infection.

### ZIKV suppresses BiP activation at the translational and transcriptional levels.

**FIG 11:**
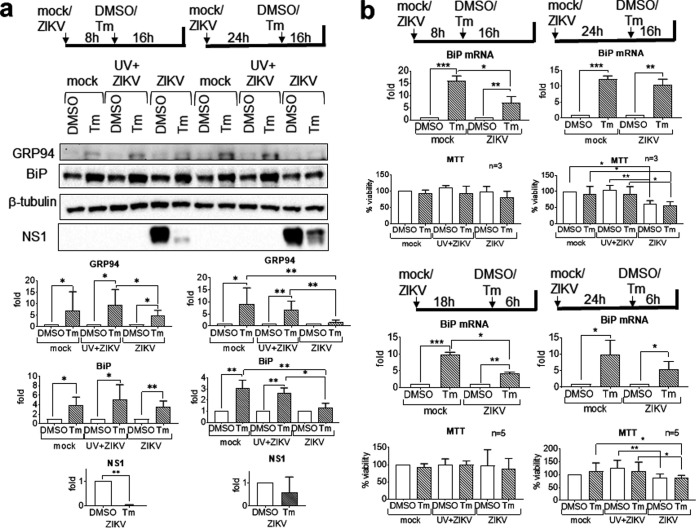
Zika virus suppresses BiP activation. A549 cells were mock infected or infected with UV-inactivated ZIKV or live ZIKV at an MOI of 1. Tunicamycin (2 μg/ml) or its DMSO solvent control was added. (a) Western blotting showing the KDEL-reactive proteins GRP94 and BiP. Presented is a representative from the same infection of three independent repeats. Protein bands were quantified, normalized against a β-tubulin internal control, and expressed as a ratio to solvent control, which was set as 1. Data on the bottom are means and SD for three independent repeats. (b) Real-time RT-qPCR of BiP mRNA. Data were normalized against an endogenous control GAPDH mRNA and are expressed as a ratio to solvent control, which is set as 1. Data are means and SD for three independent infections. Cell viability MTT data were analyzed by paired ratio *t* test and are presented as means and SD for three to five independent repeats, expressed as a percentage of DMSO-treated, mock-infected control, which was set as 100%. *, *P* < 0.05; **, *P* < 0.01; ***, *P* < 0.001.

To study whether ZIKV could actively suppress BiP activation, we infected A549 cells with ZIKV for 24 and 40 h, respectively, and treated cells with Tm for 16 h before the end of infection. A 16-h Tm treatment had been predetermined to induce robust BiP expression (Fig. 10a). ZIKV but not UV-inactivated virus infection suppressed BiP expression only when Tm was added at 24 hpi (Fig. 11a). The suppressive effect was not a combined effect of ZIKV and Tm on cell viability, because there was no significant difference in cell viability between dimethyl sulfoxide (DMSO)- and Tm-treated ZIKV-infected samples (Fig. 11b).

Because of the modest upregulation of Bip mRNA, we sought to study whether ZIKV also suppressed BiP at the transcriptional level using real-time reverse transcription-quantitative PCR (RT-qPCR). We infected A549 cells with ZIKV for different times before Tm was added for 6 h or 16 h before the end of infection (Fig. 11b). Regardless of the duration of Tm treatment, ZIKV infection significantly suppressed Tm-induced BiP transcription at 24 hpi without affecting cell viability, whereas ZIKV had no suppressive effect when Tm was added at 24 hpi. Because inhibition of BiP transcription did not lead to inhibition of BiP protein expression (compare Fig. 11a, left, and Fig. 11b, top left) and inhibition of BiP protein expression occurred in the absence of inhibition of BiP transcription (compare Fig. 11a, right, and Fig. 11b, top right), we speculate that ZIKV actively suppresses BiP translation and transcription by independent mechanisms at different time points of its life cycle.

### ZIKV is inhibited by the UPR but is refractory to UPR inhibition once infection has been established.

**FIG 12:**
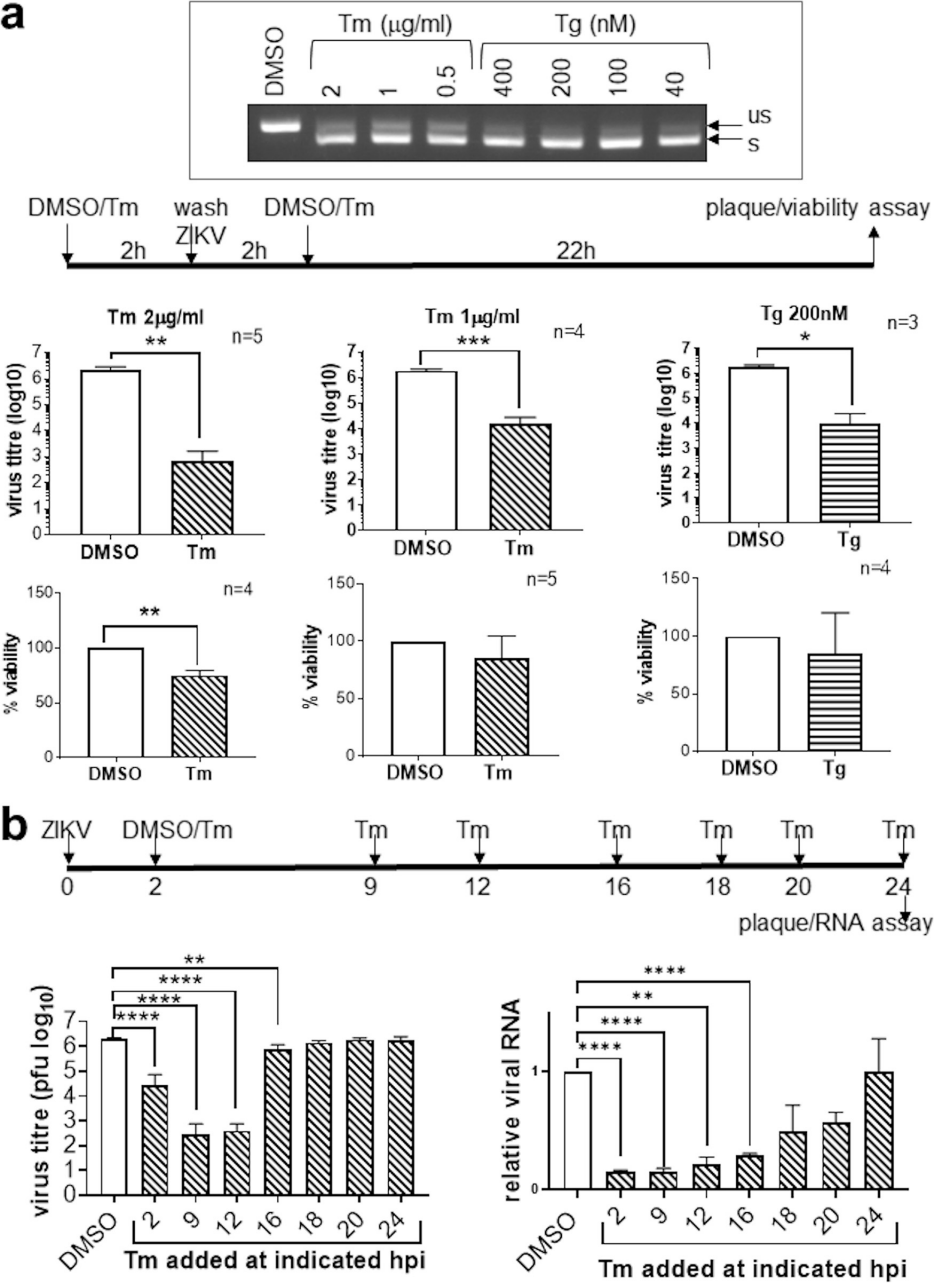
Zika virus infection is inhibited by UPR inducers but is refractory to inhibition once infection has been established. (a) A549 cells were treated with the indicated doses of tunicamycin (Tm), thapsigargin (Tg), or DMSO solvent control for 2 h. Tm/Tg was washed off, and cells were infected with ZIKV at an MOI of 1 for 2 h before Tm/Tg was added back to incubate for a further 22 h. (Top) Agarose gel electrophoresis showing XBP1 splicing in Tm- and Tg-treated samples at the indicated doses. us, unspliced; s, spliced. (Middle) Plaque assay data were analyzed by paired *t* test and are presented as means and SD. (Bottom) Cell viability data were analyzed by ratio paired *t* test and are presented as means and SD. Data are expressed as a percentage of the solvent control, which was set as 100%. The number of independent repeats (n) is indicated. (b) A549 cells were infected with ZIKV at an MOI of 1 and treated with 2 μg/ml Tm or DMSO solvent control at the indicated hours postinfection (hpi). Supernatants harvested at 24 hpi were measured for virus titers. RNA extracted from cells was quantified for viral RNA using RT-qPCR, normalized against an endogenous control (GAPDH mRNA), and expressed as a ratio to solvent control, which was set as 1. Data were analyzed by one-way ANOVA and are presented as means and SD for three independent repeats. *, *P* < 0.05; **, *P* < 0.01; ***, *P* < 0.001; ****, *P* < 0.0001.

The UPR can be pro- or antiviral (14). To find out whether the UPR was pro- or anti-ZIKV, we pre-exposed A549 cells to an ER stress inducer, Tm, for 2 h before infection. Compared to the DMSO solvent control, pre-exposure to 2 μg/ml Tm resulted in a 3.6-log reduction in virus titer at 24 hpi (Fig. 12a). This concentration of Tm also reduced cell viability by 27%, although no cell death was observed. To exclude the possibility that reduction in virus titer was a result of reduced cell viability, we used a noncytotoxic dose of 1 μg/ml Tm and still observed a 2-log reduction in virus titer compared to that of the DMSO-treated sample. Tm is also an inhibitor of protein glycosylation (48). To exclude the possibility that the antiviral effect of Tm is mediated via its inhibitory effect on viral envelope protein glycosylation rather than via the UPR, we used Tg, which induces the UPR via a completely different mechanism of inhibition of the sarcoplasmic/ER Ca^2+^ ATPase to deplete the ER Ca^2+^ store (49). Similar to Tm, a noncytotoxic dose of Tg was able to reduce virus titer by 2.3 log. All these drug concentrations had been confirmed to be capable of eliciting the UPR by their ability to induce XBP1 splicing.

To eliminate the inhibitory effects of the UPR inducers on pre-entry steps and to pinpoint the steps inhibited by the UPR, we added Tm at different time points postinfection. Because active viral translation and replication started at 16 hpi (Fig. 1), we performed a time course experiment in which 2 μg/ml Tm was added before and after 16 hpi and virus titers were measured at 24 hpi (Fig. 12b). Addition of Tm at 2 hpi resulted in a 1.9-log reduction in virus titer, indicating that Tm inhibits a postentry step. Addition of Tm at 9 and 12 hpi resulted in near abolishment of virus titers (3.8- and 3.7-log reduction), suggesting that the virus succumbs to the inhibitory effects of UPR before active virus translation and replication can ensue. In contrast, addition of Tm after 16 hpi reduced the virus titer by only 0.4 log, which was 3.3 log higher than when Tm was added at 12 hpi. Because Tm can impair protein glycosylation, which may then affect viral progeny infectivity, we used RT-qPCR to measure intracellular viral RNA in order to confirm the effects of the UPR on viral replication. Similar to the virus titer results, addition of Tm at 2, 9, and 12 hpi resulted in a dramatic reduction in viral RNA. The level of viral RNA started to recover and increase with time when Tm was added from 16 to 24 hpi. Together, these results suggest that the UPR is antiviral but ZIKV is refractory to UPR inhibition once infection has been established, indicating that ZIKV may possess an anti-UPR machinery.

## DISCUSSION

Increasing evidence suggests an important role of the UPR in the virus life cycle (14). The tripartite UPR is often skewed in virus infection as a result of a virus-host coevolutionary arms race (4, 14). Understanding virus-host UPR interaction is paramount in a targeted antiviral approach. By dissecting the kinetics of the UPR, we were able to identify an atypical tripartite UPR in ZIKV infection in which activation of the three proximal sensors is sustained whereas that of downstream effectors is transient.

Our kinetic studies showed that ZIKV translation and replication induced UPR sensor activation and transient ISR and XBP1 splicing, which in turn preceded virus particle release, suggesting a causal (rather than casual) interaction between ZIKV and the UPR. UPR can be proviral or antiviral at different steps of the virus life cycle (14). Understanding UPR kinetics is important in understanding temporal regulation of the UPR and virus-host interplay during the course of virus infection, leading to correct timing of interference in drug design. Indeed, we found that ZIKV infection was inhibited by pretreatment with chemical UPR inducers but was refractory to the inhibitory activity of chemical UPR inducers once infection had been established, suggesting that ZIKV has anti-UPR mechanisms that may be able to modulate and co-opt the UPR in its life cycle. Indeed, we showed that ZIKV continued to translate, replicate, and produce virus particles and spread in the face of a robust UPR, whereas others have shown co-option of IRE1α in ZIKV replication (22, 23). This kinetic study lays the foundation to dissect the dynamic roles of individual UPR molecules in the virus life cycle.

In this study, we identified a dichotomy in the UPR during ZIKV infection. The UPR is a homeostatic response in which activation of the proximal sensors are downregulated by the effectors in negative feedback loops (4). Previous studies have shown a role of IRE1α in facilitating ZIKV infection via XBP1 (22, 23). However, we found that sustained activation of the three sensors has been uncoupled from transient activation of UPR effectors in ZIKV infection, suggesting an independent role of the UPR sensors in ZIKV infection. Our results suggest that phospho-PERK but not phospho-eIF2α acts in synergy with the ATF6 pathway to facilitate ZIKV replication at an early phase of infection. Our results even implied a negative role of IRE1α phosphorylation on ZIKV infection. In the Kaposi’s sarcoma-associated herpesvirus, differential and sustained activation of the UPR sensor but not effector has been co-opted for lytic replication, suggesting that viruses can manipulate and repurpose the UPR pathways to aid in replication (50).

In this study, we found differential roles of the UPR in temporal regulation of ZIKV replication. Dengue virus also temporally regulate the UPR by triggering transient PERK-eIF2α phosphorylation during the early phase and IRE1α-XBP1 and ATF6 during mid-phase and late phase of infection (51). In ZIKV, phospho-PERK alone does not seem to have any effects on viral replication but may play a part in pathogenesis. Using GSK2606414, we were unable to inhibit virus yields at all three time points, in agreement with results from another group using another two PERK inhibitors, GSK2656157 and ISR1B, and PERK small interfering RNA (siPERK) (21). Injection of a PERK inhibitor rescued perturbation of neurogenesis and microcephaly in mouse brains without displaying antiviral effects (19). Our results suggest that phospho-PERK, however, exhibits a synergistic effect with the ATF6 pathway at 24 hpi and 48 hpi. The apparently low level of p50nATF6 at 24 hpi is sufficient to facilitate ZIKV replication. ATF6, on the other hand, is required for late phase of infection, as demonstrated by the significantly high degree of p90ATF6 disappearance and the ability of Ceapin-A7 to reduce virus titers at 48 and 72 hpi. However, Ceapin-A7 alone had little (24 hpi) or modest effects on virus titers (48 hpi, 72 hpi) but synergized with GSK2606414 to cause 90% (10-fold) and 66% reduction in virus titers at 24 hpi and 48 hpi. Others have reported modest inhibition of ZIKV replication in cell and mouse models using genetic and chemical inhibition of IRE1α/XBP1 (19, 21–23, 52). This indicates that ATF6 or other UPR arms are not the sole pathway modulating ZIKV infection and they may act in cooperation or in opposition. Indeed, using a chemical chaperone, 4-PBA, that inhibits the PERK and the ATF6 pathways and potentiates the IRE1α pathway, we achieved a significant 3.74-log (5,623-fold) reduction in virus titer, suggesting that potent inhibition of ZIKV infection may require synergistic manipulation of the three UPR arms. Further studies using combinatorial strategy will be required to dissect the relative importance, kinetics, and synergism of individual UPR arms in ZIKV infection as an essential step in combinatorial drug discovery.

Whereas the ISR was activated during early phase of infection, PERK-eIF2α phosphorylation was differentially upregulated during late phase of infection, indicating a functional dichotomy of PERK-eIF2α phosphorylation and ATF4-CHOP in different phases of virus infection. Normally, PERK-eIF2α phosphorylation inhibits global protein synthesis but upregulates ATF4 translation, which then transactivates GADD34 to recruit PP1 to dephosphorylate phospho-eIF2α in a negative-feedback loop (10, 11). However, under some stress conditions, such as UV irradiation, that suppress ATF4 transcription, eIF2α phosphorylation does not enhance ATF4 expression (53). In tumor cells, the ubiquitin ligase RNF4 ubiquitinates and stabilizes phospho-eIF2α but not ATF4 and CHOP, generating a positive feed-forward loop (54). Toll-like receptor engagement reversed the effects of eIF2α phosphorylation on global protein synthesis and ATF4 translation, resulting in differential suppression of ATF4-CHOP but not PERK-eIF2α phosphorylation (55). In ZIKV infection, we found that phospho-eIF2α is still functional as a translational blocker but is unable to activate ATF4 translation. We reasoned that this may be due to suppression of ATF4 transcription by ZIKV or a global shutdown of translation by rRNA degradation which is independent of eIF2α phosphorylation. ATF4 and CHOP have short half-lives (30 min to 4 h) and hence disappeared rapidly, whereas PERK has a long half-life of 13h (6, 56, 57). We hypothesize that a block in the negative feedback loop by a muted GADD34 activation and a blunted ATF4 response and concurrent activation of eIF2α kinases are essential to upregulate eIF2α phosphorylation to induce global protein synthesis inhibition and cell lysis to facilitate virus release at the late phase of infection.

The UPR is a cellular homeostatic response (4). It is therefore intriguing to detect sustained activation of the UPR sensors throughout the life cycle of ZIKV. BiP is the master negative regulator of the UPR (5, 6). It is transcriptionally activated via the ATF6 and IRE1α pathways in a negative-feedback loop to restore ER homeostasis. BiP is commonly induced in virus infections, including that of flaviviruses (58). Paradoxically, we found that BiP expression was blunted in ZIKV infection. We and others detected a low level of BiP protein expression and modest BiP transcriptional upregulation in various cell types and mouse brain infected with different ZIKV strains (20, 21, 26, 28, 29, 59). Not only was BiP expression blunted, but also, we and others found that ZIKV was able to suppress BiP upregulation by chemical UPR inducers, Tm and Tg, suggesting an antiviral role of BiP (this study and reference 26). Furthermore, our results suggest that ZIKV actively suppresses BiP translation and transcription by independent mechanisms at different time points of its life cycle, providing an important information in antiviral targeting. In contrast, inhibition of ZIKV infection by BiP depletion suggests a proviral role of BiP (29, 59). However, it remains possible that depletion of the master negative UPR regulator favors ZIKV infection by sustaining UPR sensor activation. It is, therefore, tempting to speculate that ZIKV requires a sustained UPR to complete its life cycle; hence, it is essential to blunt BiP expression. The ability of ZIKV E protein to bind BiP may suggest this. Apart from the UPR, BiP can mediate its pro-/antiviral effect via its chaperone activity. BiP facilitates ZIKV infection in human placental trophoblasts and astrocytoma cells by stabilizing interaction between the placental alkaline phosphatase and ZIKV proteins, although its significance in other cell types devoid of placental alkaline phosphatase remains undetermined (60). In contrast, BiP represses yellow fever virus infection by stabilizing the IFN-α-inducible protein 6 (61). It is therefore essential to dissect the pro-/antiviral role of BiP in ZIKV life cycle and the mechanisms of ZIKV suppression of BiP translation and transcription in enabling development of a new class of host-targeting agent.

Our results also suggest interaction between the UPR, ISR, and IFN response. GSK2606414 inhibited phospho-PERK at 24 hpi without significantly affecting phospho-eIF2α, suggesting that another ISR kinase is responsible for transient ISR at 24 hpi. The IFN-inducible PKR has been shown to be activated in ZIKV infection of A549 cells at 24, 48, and 60 hpi (25). ZIKV infection of A549 cells led to secretion of IFN-β as early as 18 hpi (34). We also detected a low level of RIDD-independent rRNA degradation at 24 hpi, which suggests activation of the IFN response. It is, therefore, likely that PERK and PKR phosphorylate eIF2α differentially and cooperatively at different steps of the ZIKV life cycle. The kinetics of host-translational shutoff, cell lysis, and virus particle production coincided with that of eIF2α phosphorylation and rRNA degradation. Surprisingly, host translational shutoff during ZIKV infection was not impeded in RNase L-knockout cells (62). Therefore, it is possible that host translational shutoff requires cooperation between rRNA degradation and eIF2α phosphorylation to prime cells for lysis and virus release. We have just begun to appreciate UPR-IFN interplay. Recently, IRE1α has been shown to prime innate immunity in tick-borne encephalitis virus in an IFN regulatory factor 3 (IRF3)-dependent and IFN-independent manner, whereas IRF3 had no effects on ZIKV infection in the absence of Tm cotreatment (63).

In conclusion, we have demonstrated an atypical tripartite UPR in ZIKV infection in which transient effector activation is associated with sustained UPR sensor activation and a blunted BiP response. ZIKV suppression of BiP expression may be necessary to blunt the BiP response to sustain UPR sensor activation to facilitate ZIKV infection. ZIKV infection is differentially regulated by the UPR molecules in a temporal manner. Phosphorylation of one of the sensors, PERK, facilitates ZIKV infection in synergy with the ATF6 pathway at early phase of infection, whereas the ATF6 pathway facilitates late phase of infection. Sustained eIF2α phosphorylation and rRNA degradation may be responsible for host translational shutoff, cell lysis, and virus release, suggesting UPR-IFN cooperation. ZIKV infection was inhibited by pretreatment of the UPR inducers but was refractory to the inhibitory activity of the UPR inducers once infection had been established, suggesting that ZIKV has anti-UPR mechanisms that may be able to modulate and co-opt the UPR in its life cycle.

## MATERIALS AND METHODS

### Cell cultures.

A549 and A549Npro human lung epithelial cells were cultured in Dulbecco’s modified Eagle’s medium with 4 mM glutamate (DMEM; Sigma) and supplemented with 10% fetal calf serum (FCS; Sigma), 100 U/ml penicillin, and 100 μg/ml streptomycin (Sigma) at 37°C and 5% CO_2_. The culture medium of A549Npro was supplemented with 2 μg/ml puromycin (Sigma). A549Npro has been transfected with the Npro gene from bovine viral diarrhea disease virus and was a kind gift from Richard Randall (St. Andrews) (64). C6/36 is derived from the larval tissue of the mosquito species Aedes albopictus and was a kind gift from Agnieszka Szemiel (Richard Elliott’s laboratory, Glasgow). C6/36 cells were cultured in Eagle’s minimal essential medium (MEM) supplemented with 1× nonessential amino acid (NEAA), 10% FCS, 100 U/ml penicillin, and 100 μg/ml streptomycin (Sigma) at 28°C and 5% CO_2_.

### Cell transfection.

Cells were transfected with 2 μg poly(I·C) using Lipofectamine 3000 (Invitrogen) according to the manufacturer’s instructions apart from omission of the P3000 reagent.

### Virus stocks.

A549Npro cells seeded in 6-well plate to 70% confluence were transfected with RNA extracted from ZIKV-infected cells (Brazilian strain PE243; a kind gift from Alain Kohl, Glasgow) using Lipofectamine MessengerMax according to the manufacturer’s instructions (Invitrogen) (65). When 70% cytopathicity was observed, the supernatant was harvested and clarified at 1,000 rpm and 4°C for 10 min before being used to infect new A549Npro cells in DMEM and 25 mM HEPES (Sigma) for 2 h at 37°C and 5% CO_2_ with periodic rocking. Postinfection medium (DMEM, 25 mM HEPES, 2% FCS) was then added, and supernatant was harvested after about 7 days, when 70% cytopathicity was observed. The supernatant was clarified at 1,000 rpm/4°C for 10 min to establish working stocks for the infection of C6/36 insect cells.

C6/36 cells were mock infected or infected with ZIKV at an MOI of 0.01 for 2 h in MEM, 1% NEAA, 15 mM HEPES (Sigma) at 28°C and 5% CO_2_ with periodic rocking. Insect postinfection medium (MEM, 1% NEAA, 15 mM HEPES supplemented with 2 or 10% FCS) was then added, and supernatant was harvested after 6 to 7 days. Supernatant harvested from mock-infected and infected cells was used in mock infection and infection. To inactivate virus, supernatant harvested from infected cells was transferred to tissue culture plates to a maximum depth of 2 mm; plates were placed on an ice box and then irradiated with a UV lamp for 1 h inside a SterilGard class II biological safety cabinet (Baker). Virus inactivation was confirmed using plaque assay of neat and diluted supernatants.

### Virus infection.

A549 cells seeded at 70 to 90% confluence were mock infected or infected with ZIKV or UV-inactivated ZIKV harvested from C6/36 cells at an MOI of 1 in DMEM, 25 mM HEPES for 2h at 37°C and 5% CO_2_ with periodic rocking. After 2 h, the virus was removed, washed once with phosphate-buffered saline (PBS), and replaced with DMEM, 25 mM HEPES, and 10% FCS.

### Plaque assay.

A549Npro cells were seeded at 3 × 10^5^ per well of a 12-well plate and infected with 200 μl of serial dilutions of virus stocks, in duplicate, in DMEM, 15 mM HEPES for 2 h at 37°C and 5% CO_2_ with periodic rocking. The virus was removed and washed once with PBS before 1 ml of 0.7% agarose or 2% carboxymethylcellulose in DMEM, 4 mM glutamate, 25 mM HEPES, 0.24% NaHCO_3_, and 2% FCS was added to each well. The plate was incubated at 37°C and 5% CO_2_ for 6 days. Plaques were visualized by incubating with 5 mg/ml 3,4,5 dimethylthiazol-2-yl-2,5, diphenyltetrazolium bromide (MTT) (Sigma/Fisher) overnight or fixed with 10% formaldehyde for 1 h followed by staining with 1% crystal violet for 30 min.

### Western blotting.

Protein lysates were harvested into radioimmunoprecipitation assay buffer (RIPA) buffer (50 mM Tris [pH 8.0], 150 mM NaCl, 1% NP-40, 0.5% Na deoxycholate, 0.1% SDS) plus protease inhibitors (Sigma) or SDS buffer (4% SDS, 125 mM Tris pH 6.8) plus protease and phosphatase inhibitors (Sigma). Protein concentrations were determined by bicinchoninic acid (BCA) assay (Sigma). Equal amounts of proteins were separated on TGX stain-free SDS-PAGE gels (Bio-Rad), transferred to polyvinylidene difluoride membranes (Millipore), and blocked in 5% semiskim milk (Marvel) in 0.1% Tween 20 (Sigma)–Tris-buffered saline (TBS) (50 mM Tris [pH 7.4], 150 mM NaCl) before being probed against primary and horseradish peroxidase (HRP)-conjugated secondary antibodies in the same buffer. For detection of p-eIF2α, membrane was blocked in 3% bovine serum albumin (BSA) (Sigma) in 0.1% Tween 20–TBS and the anti-p-eIF2α antibody and secondary antibody were incubated in 0.1% Tween 20–TBS. Protein bands were detected using Clarity enhanced chemiluminescence (ECL) substrate (Bio-Rad). Images were captured and quantified using a ChemiDoc XRS+ system (Bio-Rad) and ImageLab 6.0.1 software (Bio-Rad). Quantified data were derived from low-exposure bands. Antibodies were used at the following concentrations: PERK, CHOP, p-eIF2α, and eIF2α, 1:1,000 (Cell Signaling Technology); p-IRE1 and IRE1, 1:1,000 (Novus Biologicals); ATF6 and ATF4, 1:1,000 (BioLegend); KDEL, 1:5,000 (Stressgen); ZIKV NS1, 1:1,000 (Abcam); β-actin, 1:5,000 (Bioss); and β-tubulin, 1:5,000 (Sigma). Secondary anti-rabbit immunoglobulin antibody (Cell Signaling Technology) was used at 1:1,000 for PERK, p-eIF2α, eIF2α, p-IRE1, IRE1, and β-actin. Secondary anti-mouse immunoglobulin antibody (Cell Signaling Technology) was used at 1:1,000 for CHOP, NS1, and β-tubulin. Secondary anti-rat immunoglobulin antibody (BioLegend) was used at 1:1,000 for ATF6 and ATF4. Some of the blots were stripped and reprobed as previously described (66, 67).

### RT-PCR and RT-qPCR.

RNA was extracted using either the RNA extraction buffers TRIzol (Invitrogen), RNA Bee (Amsbio), and RNAzol RT (Sigma) or an Isolate II RNA minikit (Bioline) according to the manufacturers’ instructions. RT-PCR was performed as described previously (68). Each primer pair was designed with at least one of them spanning the exon-intron boundary so that mRNA but not genomic DNA was amplified (Table 1) (66, 69). The cycling parameters were initial denaturation at 94°C for 2 min followed by 25 to 40 cycles of denaturation at 94°C for 10s, annealing at 50 to 55°C for 30 s, and elongation at 68°C for 45 s with a final extension at 68°C for 7 min. PCR band intensity was measured using ImageJ (NIH) or ImageLab 6.0.1 (Bio-Rad) and normalized against an internal control, glyceraldehyde 3-phosphate dehydrogenase (GAPDH). Low-exposure gels were used in image quantification.

**TABLE 1:** Primers used in RT-PCR and RT-qPCR

Primer name	Sequence
XBP1-sense	5′-GCTGAGGAGGAAACTGAAAAAC-3′
XBP1-antisense	5′-TGCCCAACAGGATATCAGAC-3′
ZIKV-sense	5′-TTCCATTACCTTGGCACGCT-3′
ZIKV-antisense	5′-TTTTGGCATGTGCGTCCTTG-3′
CHOP-sense	5′-TGAGGAGAGAGTGTTCAAGAAG-3′
CHOP-antisense	5′-TCCAGGAGGTGAAACATAGG-3′
BiP-sense	5′-GCCGTTCAAGGTGGTTGAAA-3′
BiP-antisense	5′-CCAAATAAGCCTCAGCGGTT-3′
hEDEM1-sense	5′-ACAACTACATGGCTCACGCC-3′
hEDEM1-antisense	5′-AGATTTGAAGGGTCCCCGC-3′
GAPDH-sense	5′-CCTGTTCGACAGTCAGCCG-3′
GAPDH-antisense	5′-CGACCAAATCCGTTGACTCC-3′

Real-time RT-qPCR was performed using 1/50 of the RT reaction mixture in Power SYBR green PCR master mix (Applied Biosystems) and 300 nM sense and anti-sense primers (Table 1). RT-qPCR was run on a StepOnePlus system (Applied Biosystems) using 10 s denaturation at 95°C followed by 40 cycles of 95°C for 15 s and 60°C for 60 s; melting curves were generated using 95°C for 15 s, 60°C for 60 s, and 95°C for 15 s. Data were analyzed by the 2^−ΔΔ^*^CT^* method using GAPDH as an endogenous control.

### Cell viability assay.

Cells seeded at 16,000 cells per well of a 96-well plate were treated or infected. Cell viability was measured by addition of 20 μl of 5 mg/ml MTT (Sigma/Fisher) in culture medium to each well for 4h at 37°C and 5% CO_2_. MTT was removed, and 100 μl of acidified isopropanol was added to each well for 1 h to dissolve the MTT. Color was read at 570 nm with a reference at 690 nm using a plate reader (Bio-Tek Synergy HT).

### Percentage of infection and immunocytochemistry.

Cells seeded at 5.7 × 10^4^ in 8-well chamber slide (Permanox; Nunc) were mock infected or infected with ZIKV at an MOI of 1. Immunocytochemistry was performed as previously described using 1:200 anti-flavivirus group E antigen antibody (clone 4G2; Millipore) or an isotypic control and 1:500 HRP-conjugated anti-mouse immunoglobulin secondary antibody (Cell Signaling Technology) (68). Stained cells representing infected cells were counted. The percent infection in live cells was calculated as the number of stained cells over total number of cells counted. Percent infection in live and dead cells was calculated as [(% viability at 24 h − % viability at 48/72 h) × 100 + (% viability at 48/72 h × % of infection in live cells at 48/72 h)]/% viability at 24 h.

### Measurement of *de novo* protein synthesis using SUnSET.

*De novo* protein synthesis was measured using surface sensing of translation (SUnSET) (70). A549 cells seeded at 5.4 × 10^5^ cells per well of a 6-well plate were mock infected or infected with UV-inactivated or live ZIKV for 24, 48, and 72 h. As a positive control, cells were treated with 20 μg/ml cycloheximide or its ethanol solvent control for 3 h. Puromycin (10 μg/ml) was added to culture medium an hour before harvesting in RIPA buffer. Equal amounts of proteins were separated on TGX stain-free gels (Bio-Rad) by SDS-PAGE and probed against 1:1,000 antipuromycin antibody (clone 12D10; Millipore) followed by 1:1,000 HRP-conjugated anti-mouse immunoglobulin secondary antibody (Cell Signaling Technology). The total intensity of puromycin bands in each lane was quantified using ImageLab 6.0.1 software and normalized against total protein on the TGX stain-free gel. Percentage of protein synthesis was calculated as a ratio of normalized puromycin band intensity in infected or UV-inactivated virus samples to their respective mock-infected controls at the same time point. Percentage of normalized protein synthesis in the cycloheximide-treated sample is expressed as a ratio to that of an ethanol solvent control.

### Statistical analysis.

Statistical analysis was performed and graphs were plotted using Prism 8.0/9.0 (GraphPad). A ratio paired *t* test was used for the analysis of Western blots, RT-PCR, RT-qPCR, the 28S and 18S rRNA bands, and percent protein synthesis from the SUnSET data. Two-way analysis of variance (ANOVA) and Tukey’s *post hoc* test were used to analyze percent p50nATF6. A ratio paired *t* test or one-way/two-way ANOVA with Tukey’s *post hoc* test was used for the analysis of cell viability data, as specified in the figure legends. Paired *t* test or one-way ANOVA with Tukey’s *post hoc* test was used for the analysis of plaque assay data, as specified in the figure legends. One-way ANOVA with Tukey’s *post hoc* test was used for the analysis of percent infection. A Shapiro-Wilk normality test and one-sample *t* test were used for the analysis of XBP1 splicing and the degraded rRNA band against a theoretical mean of 0. A *P* value of <0.05 was considered statistically significant.
