# Erratum for Mufrrih et al., “Zika Virus Induces an Atypical Tripartite Unfolded Protein Response with Sustained Sensor and Transient Effector Activation and a Blunted BiP Response”

**DOI:** 10.1128/mSphere.00876-21

**Published:** 2021-11-03

**Authors:** Mohammed Mufrrih, Biyao Chen, Shiu-Wan Chan

**Affiliations:** a Faculty of Biology, Medicine and Health, School of Biological Sciences, The University of Manchester, Manchester, United Kingdom

## ERRATUM

Volume 6, no. 3, e00361-21, 2021, https://doi.org/10.1128/mSphere.00361-21. The following statement should be added to the Acknowledgments section of this paper: “CRediT roles for this study are as follows: Mohammed Mufrrih: Investigation; Biyao Chen: Formal analysis, Investigation; Shiu-Wan Chan: Conceptualization, Data curation, Formal analysis, Investigation, Methodology, Project administration, Resources, Supervision, Validation, Visualization, Writing - original draft, Writing - review & editing.”

